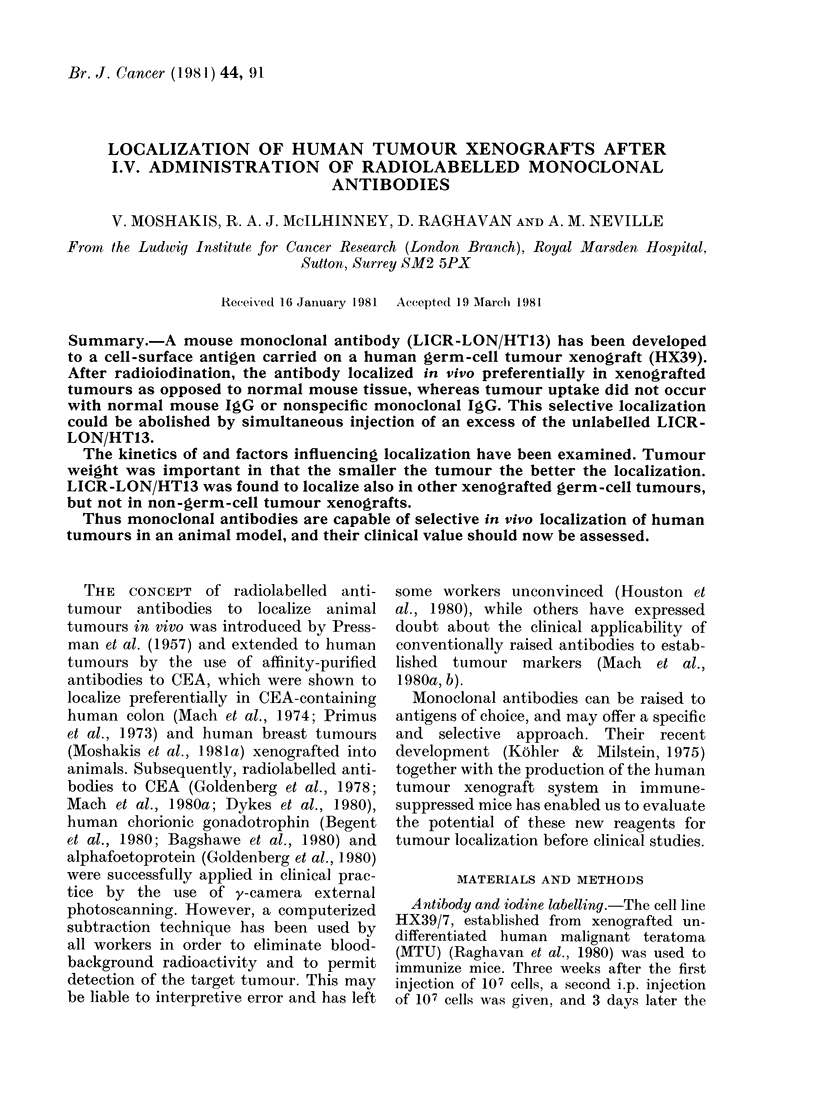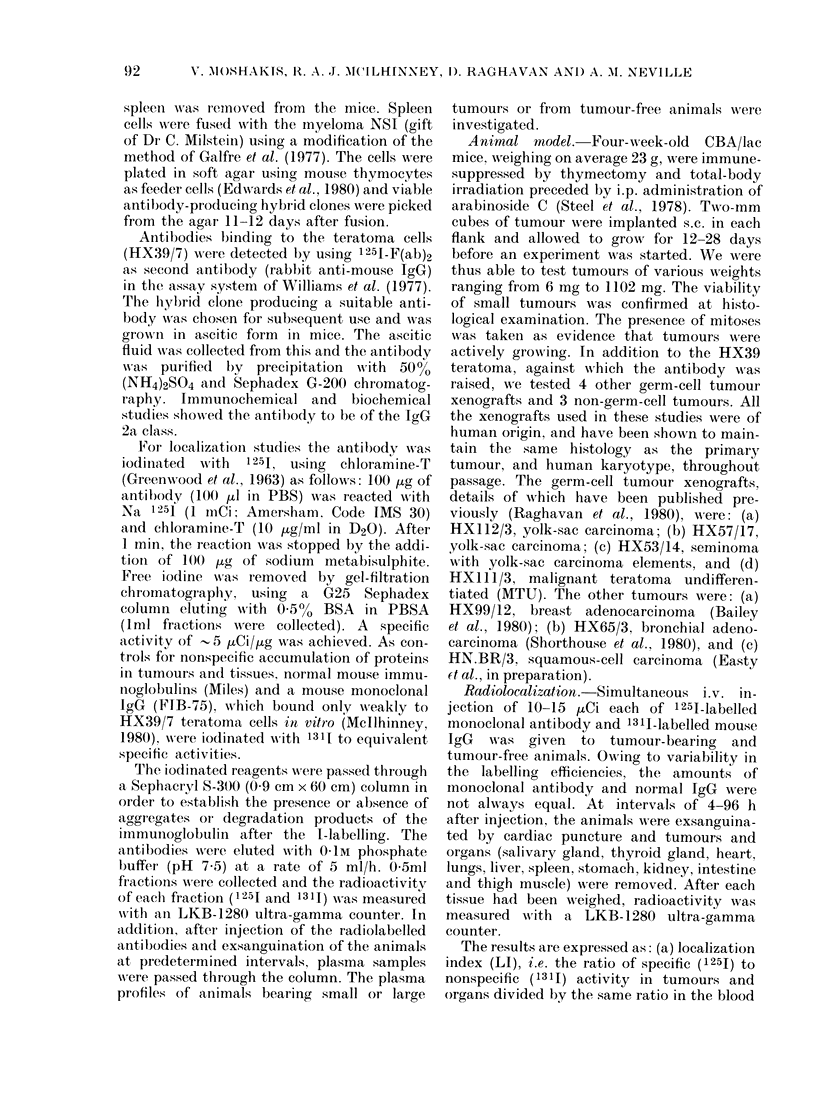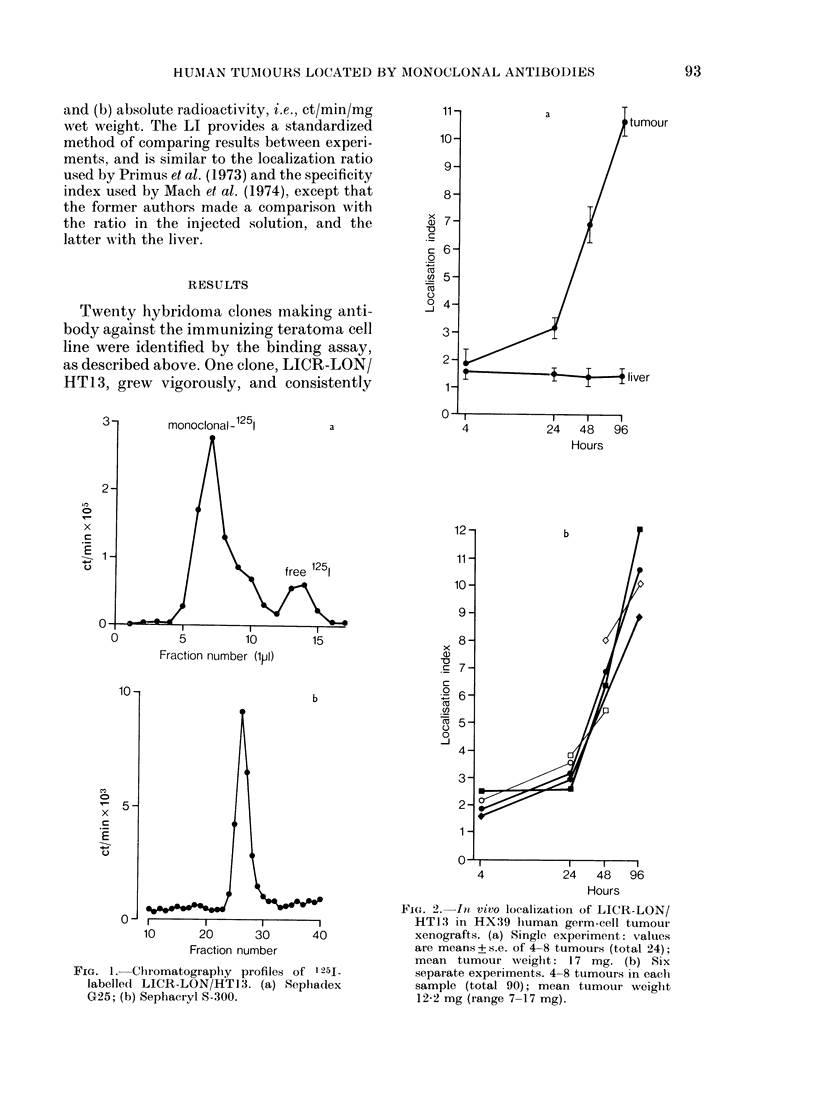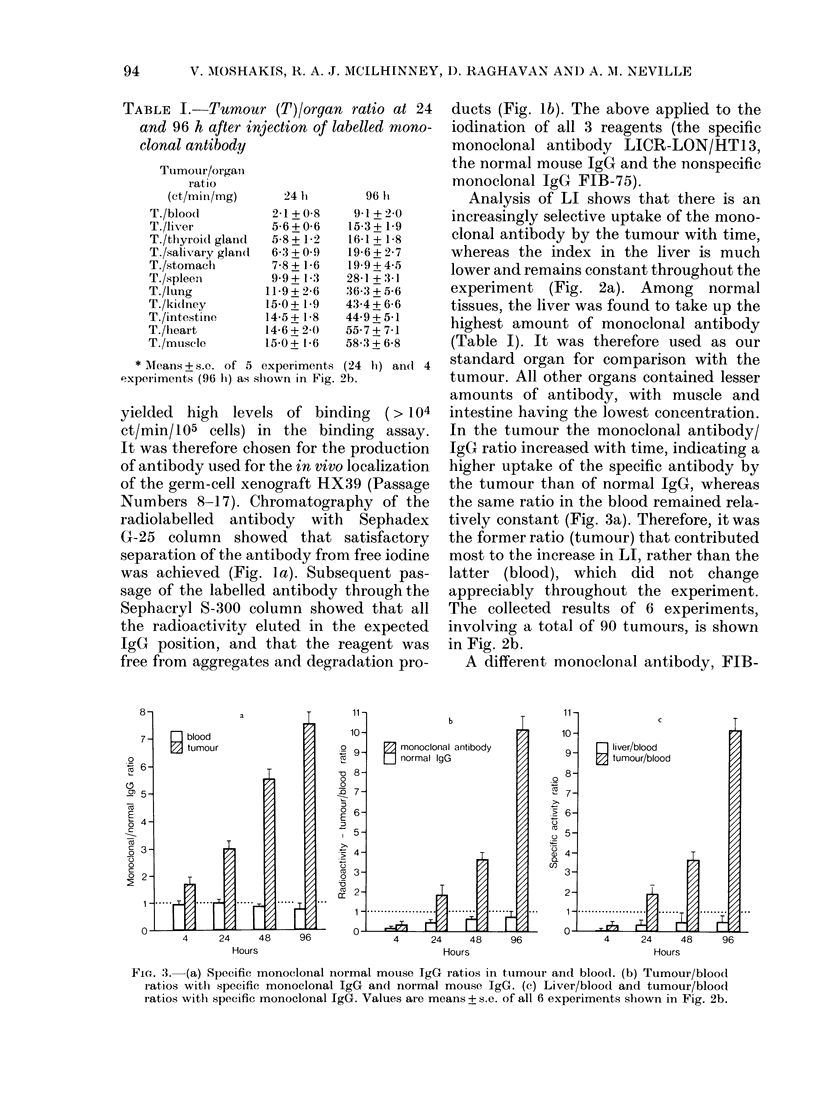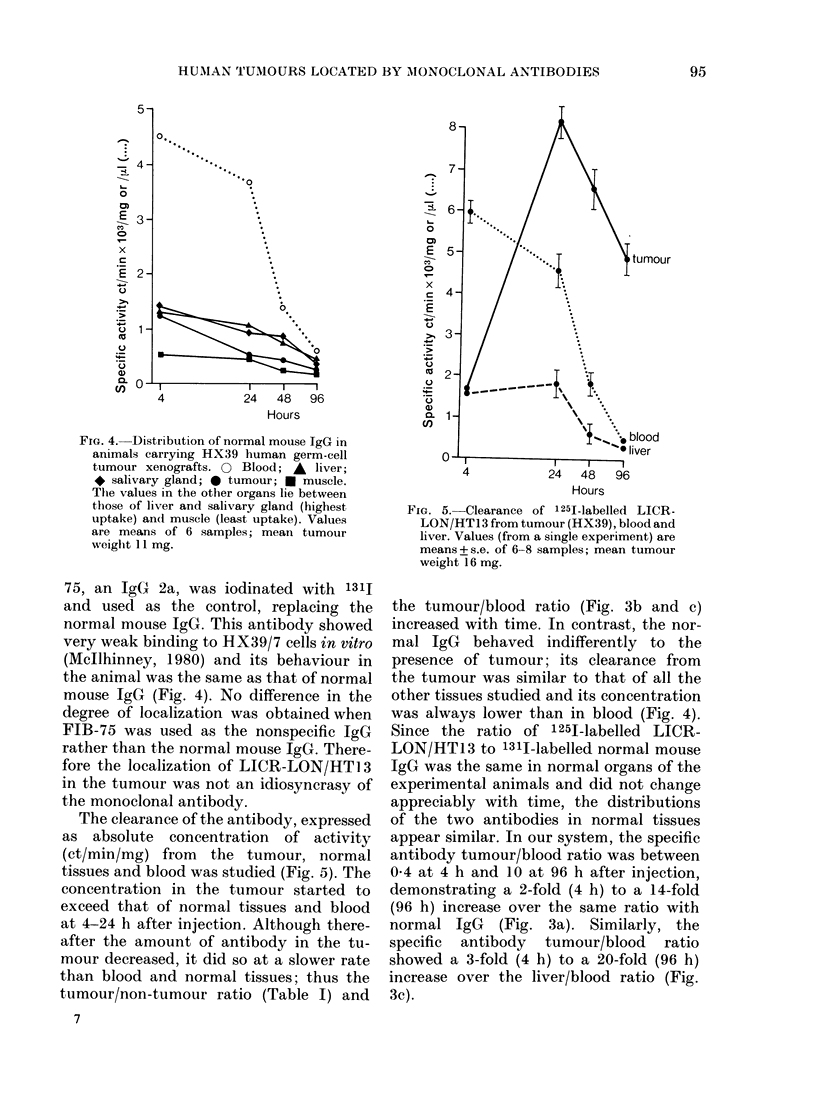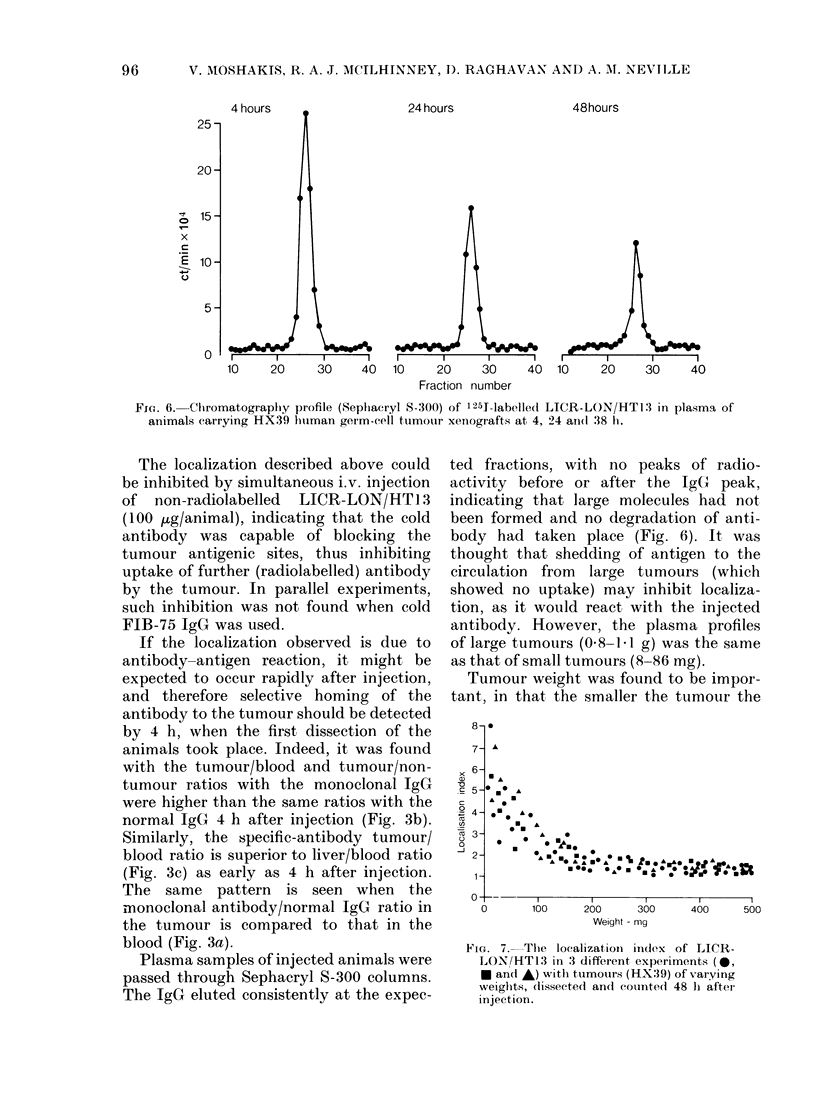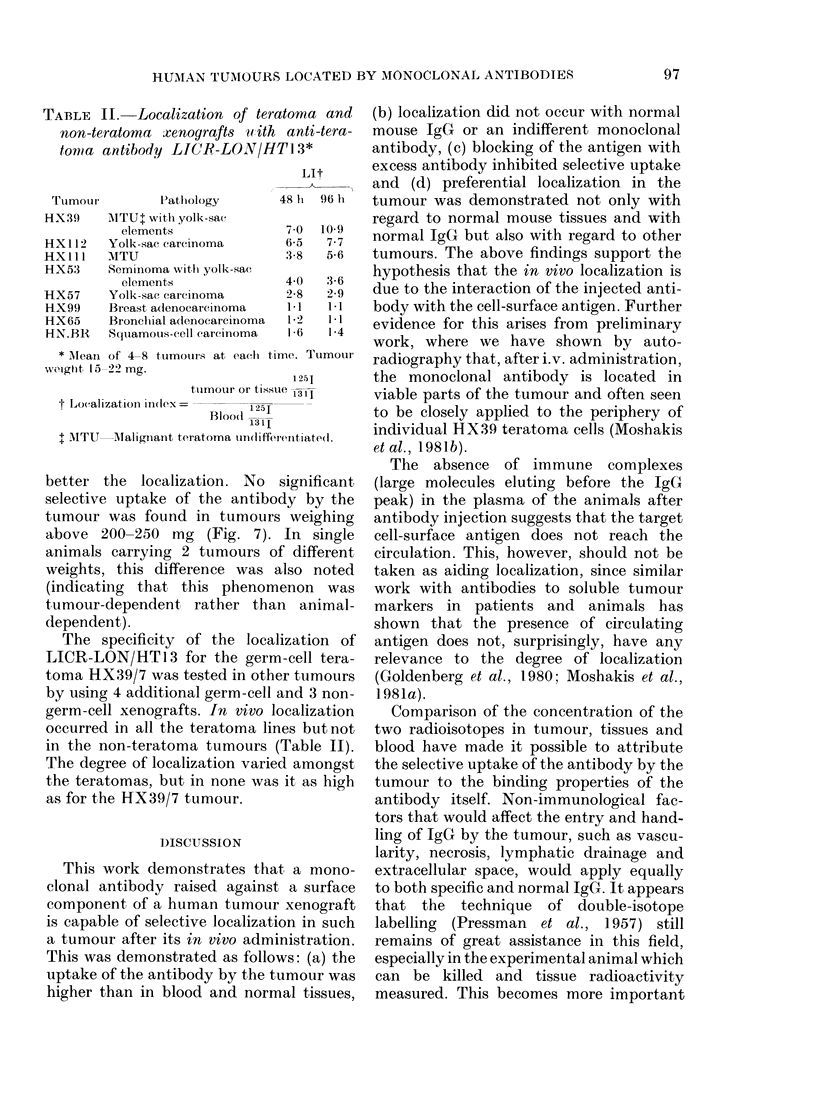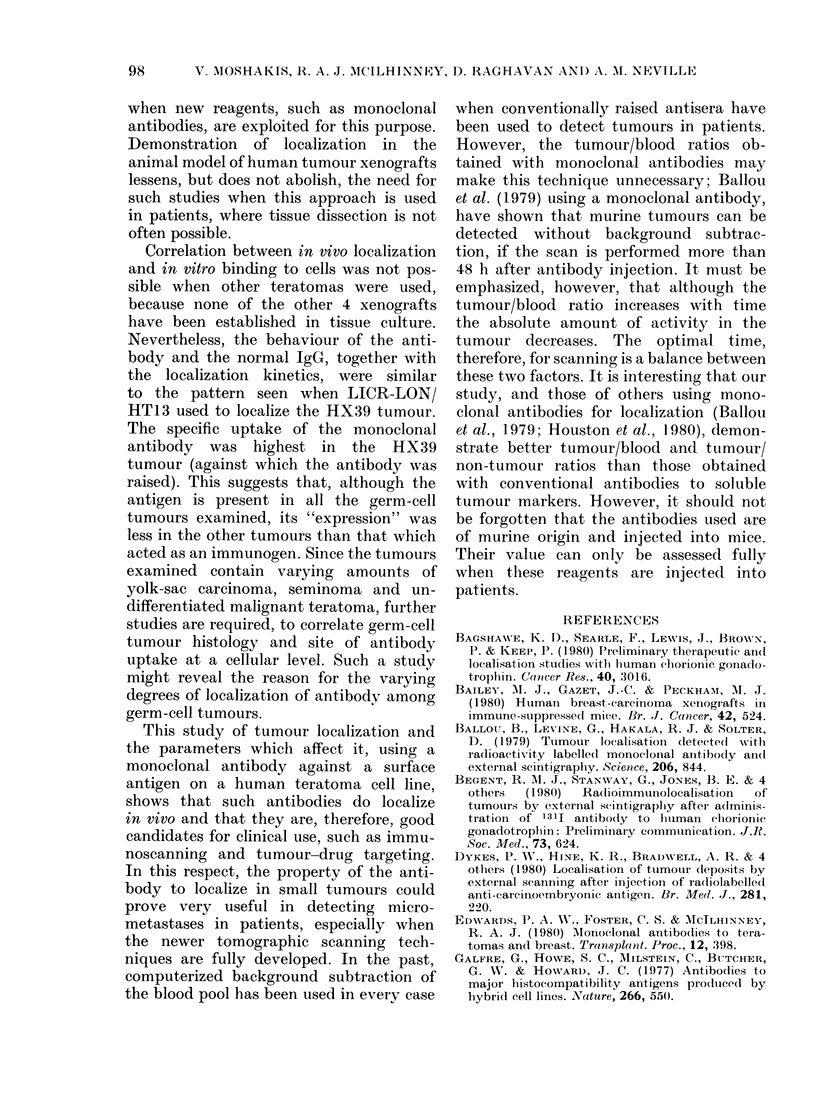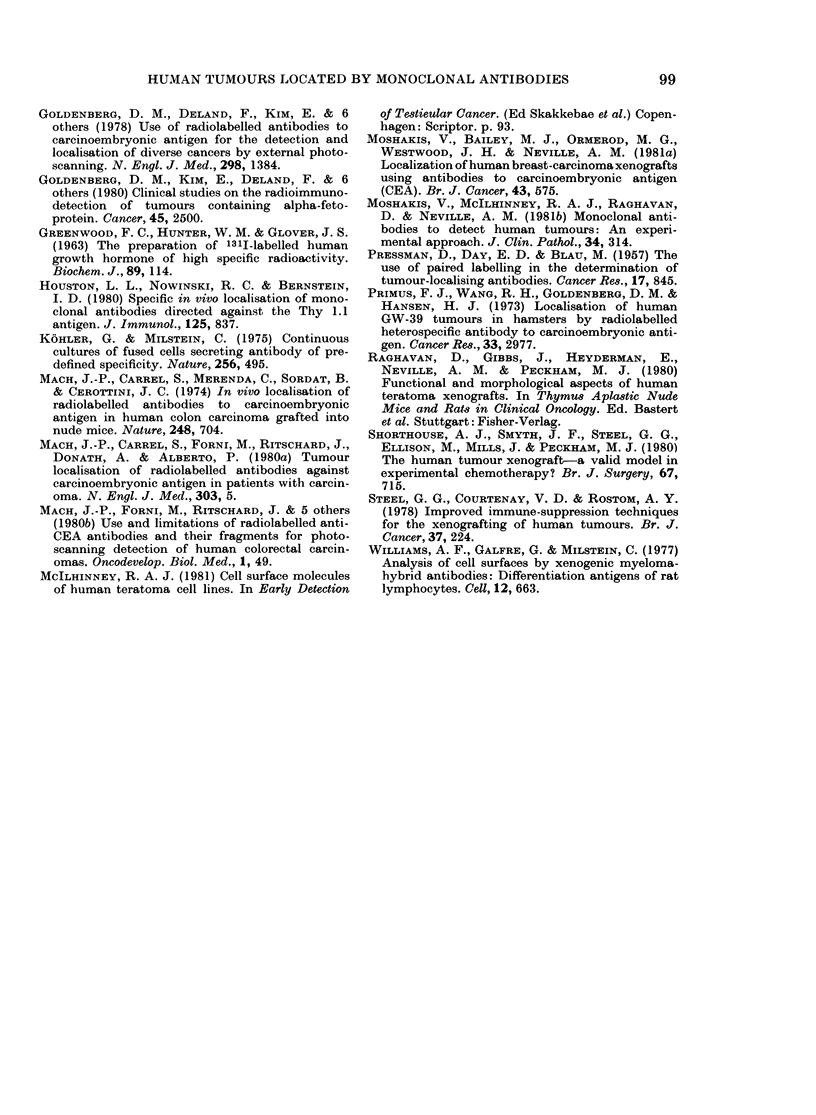# Localization of human tumour xenografts after i.v. administration of radiolabeled monoclonal antibodies.

**DOI:** 10.1038/bjc.1981.153

**Published:** 1981-07

**Authors:** V. Moshakis, R. A. McIlhinney, D. Raghavan, A. M. Neville

## Abstract

A mouse monoclonal antibody (LICR-LON/HT13) has been developed to a cell-surface antigen carried on a human germ-cell tumour xenograft (HX39). After radioiodination, the antibody localized in vivo preferentially in xenografted tumours as opposed to normal mouse tissue, whereas tumor uptake did not occur with normal mouse IgG or nonspecific monoclonal IgG. This selective localization could be abolished by simultaneous injection of an excess of the unlabelled LICR-LON/HT13. The kinetics of and factors influencing localization have been examined. Tumour weight was important in that the smaller the tumour the better the localization. LICR-LON/HT13 was found to localize also in other xenografted germ-cell tumours, but not in non-germ-cell tumour xenografts. Thus monoclonal antibodies are capable of selective in vivo localization of human tumours in an animal model, and their clinical value should now be assessed.


Br. J. Cancer (1981) 44, 91

### LOCALIZATION OF HUMAN TUMOUR XENOGRAFTS AFTER I.V. ADMINISTRATION OF RADIOLABELLED MONOCLONAL ANTIBODIES

V. MOSHAKIS, R. A. J. McILHINNEY, D. RAGHAVAN AND A. M. NEVILLE

*From the Ludwig Institute for Cancer Research (London Branch), Royal Marsden Hospital, Sutton, Surrey SM2 5PX*

Received 16 January 1981 Accepted 19 March 1981

**Summary.—A mouse monoclonal antibody (LICR-LON/HT13) has been developed to a cell-surface antigen carried on a human germ-cell tumour xenograft (HX39). After radioiodination, the antibody localized *in vivo* preferentially in xenografted tumours as opposed to normal mouse tissue, whereas tumour uptake did not occur with normal mouse IgG or nonspecific monoclonal IgG. This selective localization could be abolished by simultaneous injection of an excess of the unlabelled LICR-LON/HT13.**

**The kinetics of and factors influencing localization have been examined. Tumour weight was important in that the smaller the tumour the better the localization. LICR-LON/HT13 was found to localize also in other xenografted germ-cell tumours, but not in non-germ-cell tumour xenografts.**

**Thus monoclonal antibodies are capable of selective *in vivo* localization of human tumours in an animal model, and their clinical value should now be assessed.**

THE CONCEPT of radiolabelled anti-tumour antibodies to localize animal tumours *in vivo* was introduced by Pressman *et al.* (1957) and extended to human tumours by the use of affinity-purified antibodies to CEA, which were shown to localize preferentially in CEA-containing human colon (Mach *et al.*, 1974; Primus *et al.*, 1973) and human breast tumours (Moshakis *et al.*, 1981*a*) xenografted into animals. Subsequently, radiolabelled antibodies to CEA (Goldenberg *et al.*, 1978; Mach *et al.*, 1980*a*; Dykes *et al.*, 1980), human chorionic gonadotrophin (Begent *et al.*, 1980; Bagshawe *et al.*, 1980) and alphafoetoprotein (Goldenberg *et al.*, 1980) were successfully applied in clinical practice by the use of $\gamma$-camera external photoscanning. However, a computerized subtraction technique has been used by all workers in order to eliminate blood-background radioactivity and to permit detection of the target tumour. This may be liable to interpretive error and has left some workers unconvinced (Houston *et al.*, 1980), while others have expressed doubt about the clinical applicability of conventionally raised antibodies to established tumour markers (Mach *et al.*, 1980*a*, *b*).

Monoclonal antibodies can be raised to antigens of choice, and may offer a specific and selective approach. Their recent development (Köhler & Milstein, 1975) together with the production of the human tumour xenograft system in immune-suppressed mice has enabled us to evaluate the potential of these new reagents for tumour localization before clinical studies.

### MATERIALS AND METHODS

*Antibody and iodine labelling.*—The cell line HX39/7, established from xenografted undifferentiated human malignant teratoma (MTU) (Raghavan *et al.*, 1980) was used to immunize mice. Three weeks after the first injection of $10^7$ cells, a second i.p. injection of $10^7$ cells was given, and 3 days later the spleen was removed from the mice. Spleen cells were fused with the myeloma NSI (gift of Dr C. Milstein) using a modification of the method of Galfre *et al.* (1977). The cells were plated in soft agar using mouse thymocytes as feeder cells (Edwards *et al.*, 1980) and viable antibody-producing hybrid clones were picked from the agar 11–12 days after fusion.

Antibodies binding to the teratoma cells (HX39/7) were detected by using $^{125}$I-F(ab)$_2$ as second antibody (rabbit anti-mouse IgG) in the assay system of Williams *et al.* (1977). The hybrid clone producing a suitable antibody was chosen for subsequent use and was grown in ascitic form in mice. The ascitic fluid was collected from this and the antibody was purified by precipitation with 50% $(NH_4)_2SO_4$ and Sephadex G-200 chromatography. Immunochemical and biochemical studies showed the antibody to be of the IgG 2a class.

For localization studies the antibody was iodinated with $^{125}$I, using chloramine-T (Greenwood *et al.*, 1963) as follows: 100 $\mu$g of antibody (100 $\mu$l in PBS) was reacted with Na $^{125}$I (1 mCi; Amersham, Code IMS 30) and chloramine-T (10 $\mu$g/ml in $D_2O$). After 1 min, the reaction was stopped by the addition of 100 $\mu$g of sodium metabisulphite. Free iodine was removed by gel-filtration chromatography, using a G25 Sephadex column eluting with 0·5% BSA in PBSA (1ml fractions were collected). A specific activity of $\sim$5 $\mu$Ci/$\mu$g was achieved. As controls for nonspecific accumulation of proteins in tumours and tissues, normal mouse immunoglobulins (Miles) and a mouse monoclonal IgG (FIB-75), which bound only weakly to HX39/7 teratoma cells *in vitro* (McIlhinney, 1980), were iodinated with $^{131}$I to equivalent specific activities.

The iodinated reagents were passed through a Sephacryl S-300 (0·9 cm × 60 cm) column in order to establish the presence or absence of aggregates or degradation products of the immunoglobulin after the I-labelling. The antibodies were eluted with 0·1M phosphate buffer (pH 7·5) at a rate of 5 ml/h. 0·5ml fractions were collected and the radioactivity of each fraction ($^{125}$I and $^{131}$I) was measured with an LKB-1280 ultra-gamma counter. In addition, after injection of the radiolabelled antibodies and exsanguination of the animals at predetermined intervals, plasma samples were passed through the column. The plasma profiles of animals bearing small or large tumours or from tumour-free animals were investigated.

*Animal model.*—Four-week-old CBA/lac mice, weighing on average 23 g, were immune-suppressed by thymectomy and total-body irradiation preceded by i.p. administration of arabinoside C (Steel *et al.*, 1978). Two-mm cubes of tumour were implanted s.c. in each flank and allowed to grow for 12–28 days before an experiment was started. We were thus able to test tumours of various weights ranging from 6 mg to 1102 mg. The viability of small tumours was confirmed at histological examination. The presence of mitoses was taken as evidence that tumours were actively growing. In addition to the HX39 teratoma, against which the antibody was raised, we tested 4 other germ-cell tumour xenografts and 3 non-germ-cell tumours. All the xenografts used in these studies were of human origin, and have been shown to maintain the same histology as the primary tumour, and human karyotype, throughout passage. The germ-cell tumour xenografts, details of which have been published previously (Raghavan *et al.*, 1980), were: (a) HX112/3, yolk-sac carcinoma; (b) HX57/17, yolk-sac carcinoma; (c) HX53/14, seminoma with yolk-sac carcinoma elements, and (d) HX111/3, malignant teratoma undifferentiated (MTU). The other tumours were: (a) HX99/12, breast adenocarcinoma (Bailey *et al.*, 1980); (b) HX65/3, bronchial adenocarcinoma (Shorthouse *et al.*, 1980), and (c) HN.BR/3, squamous-cell carcinoma (Easty *et al.*, in preparation).

*Radiolocalization.*—Simultaneous i.v. injection of 10–15 $\mu$Ci each of $^{125}$I-labelled monoclonal antibody and $^{131}$I-labelled mouse IgG was given to tumour-bearing and tumour-free animals. Owing to variability in the labelling efficiencies, the amounts of monoclonal antibody and normal IgG were not always equal. At intervals of 4–96 h after injection, the animals were exsanguinated by cardiac puncture and tumours and organs (salivary gland, thyroid gland, heart, lungs, liver, spleen, stomach, kidney, intestine and thigh muscle) were removed. After each tissue had been weighed, radioactivity was measured with a LKB-1280 ultra-gamma counter.

The results are expressed as: (a) localization index (LI), *i.e.* the ratio of specific ($^{125}$I) to nonspecific ($^{131}$I) activity in tumours and organs divided by the same ratio in the blood and (b) absolute radioactivity, *i.e.*, ct/min/mg wet weight. The LI provides a standardized method of comparing results between experiments, and is similar to the localization ratio used by Primus *et al.* (1973) and the specificity index used by Mach *et al.* (1974), except that the former authors made a comparison with the ratio in the injected solution, and the latter with the liver.

### RESULTS

Twenty hybridoma clones making antibody against the immunizing teratoma cell line were identified by the binding assay, as described above. One clone, LICR-LON/HT13, grew vigorously, and consistently

FIG. 1.—Chromatography profiles of $^{125}$I-labelled LICR-LON/HT13. (a) Sephadex G25; (b) Sephacryl S-300.

FIG. 2.—*In vivo* localization of LICR-LON/HT13 in HX39 human germ-cell tumour xenografts. (a) Single experiment: values are means ± s.e. of 4–8 tumours (total 24); mean tumour weight: 17 mg. (b) Six separate experiments. 4–8 tumours in each sample (total 90); mean tumour weight 12·2 mg (range 7–17 mg).

TABLE I.—*Tumour (T)/organ ratio at 24 and 96 h after injection of labelled monoclonal antibody*

| Tumour/organ ratio (ct/min/mg) | 24 h | 96 h |
|---|---|---|
| T./blood | 2·1 ± 0·8 | 9·1 ± 2·0 |
| T./liver | 5·6 ± 0·6 | 15·3 ± 1·9 |
| T./thyroid gland | 5·8 ± 1·2 | 16·1 ± 1·8 |
| T./salivary gland | 6·3 ± 0·9 | 19·6 ± 2·7 |
| T./stomach | 7·8 ± 1·6 | 19·9 ± 4·5 |
| T./spleen | 9·9 ± 1·3 | 28·1 ± 3·1 |
| T./lung | 11·9 ± 2·6 | 36·3 ± 5·6 |
| T./kidney | 15·0 ± 1·9 | 43·4 ± 6·6 |
| T./intestine | 14·5 ± 1·8 | 44·9 ± 5·1 |
| T./heart | 14·6 ± 2·0 | 55·7 ± 7·1 |
| T./muscle | 15·0 ± 1·6 | 58·3 ± 6·8 |

* Means ± s.e. of 5 experiments (24 h) and 4 experiments (96 h) as shown in Fig. 2b.

yielded high levels of binding ($>10^4$ ct/min/$10^5$ cells) in the binding assay. It was therefore chosen for the production of antibody used for the *in vivo* localization of the germ-cell xenograft HX39 (Passage Numbers 8–17). Chromatography of the radiolabelled antibody with Sephadex G-25 column showed that satisfactory separation of the antibody from free iodine was achieved (Fig. 1*a*). Subsequent passage of the labelled antibody through the Sephacryl S-300 column showed that all the radioactivity eluted in the expected IgG position, and that the reagent was free from aggregates and degradation products (Fig. 1*b*). The above applied to the iodination of all 3 reagents (the specific monoclonal antibody LICR-LON/HT13, the normal mouse IgG and the nonspecific monoclonal IgG FIB-75).

Analysis of LI shows that there is an increasingly selective uptake of the monoclonal antibody by the tumour with time, whereas the index in the liver is much lower and remains constant throughout the experiment (Fig. 2a). Among normal tissues, the liver was found to take up the highest amount of monoclonal antibody (Table I). It was therefore used as our standard organ for comparison with the tumour. All other organs contained lesser amounts of antibody, with muscle and intestine having the lowest concentration. In the tumour the monoclonal antibody/IgG ratio increased with time, indicating a higher uptake of the specific antibody by the tumour than of normal IgG, whereas the same ratio in the blood remained relatively constant (Fig. 3a). Therefore, it was the former ratio (tumour) that contributed most to the increase in LI, rather than the latter (blood), which did not change appreciably throughout the experiment. The collected results of 6 experiments, involving a total of 90 tumours, is shown in Fig. 2b.

A different monoclonal antibody, FIB-

FIG. 3.—(a) Specific monoclonal normal mouse IgG ratios in tumour and blood. (b) Tumour/blood ratios with specific monoclonal IgG and normal mouse IgG. (c) Liver/blood and tumour/blood ratios with specific monoclonal IgG. Values are means ± s.e. of all 6 experiments shown in Fig. 2b.

FIG. 4.—Distribution of normal mouse IgG in animals carrying HX39 human germ-cell tumour xenografts. ○ Blood; ▲ liver; ◆ salivary gland; ● tumour; ■ muscle. The values in the other organs lie between those of liver and salivary gland (highest uptake) and muscle (least uptake). Values are means of 6 samples; mean tumour weight 11 mg.

FIG. 5.—Clearance of $^{125}$I-labelled LICR-LON/HT13 from tumour (HX39), blood and liver. Values (from a single experiment) are means ± s.e. of 6–8 samples; mean tumour weight 16 mg.

75, an IgG 2a, was iodinated with $^{131}$I and used as the control, replacing the normal mouse IgG. This antibody showed very weak binding to HX39/7 cells *in vitro* (McIlhinney, 1980) and its behaviour in the animal was the same as that of normal mouse IgG (Fig. 4). No difference in the degree of localization was obtained when FIB-75 was used as the nonspecific IgG rather than the normal mouse IgG. Therefore the localization of LICR-LON/HT13 in the tumour was not an idiosyncrasy of the monoclonal antibody.

The clearance of the antibody, expressed as absolute concentration of activity (ct/min/mg) from the tumour, normal tissues and blood was studied (Fig. 5). The concentration in the tumour started to exceed that of normal tissues and blood at 4–24 h after injection. Although thereafter the amount of antibody in the tumour decreased, it did so at a slower rate than blood and normal tissues; thus the tumour/non-tumour ratio (Table I) and the tumour/blood ratio (Fig. 3b and c) increased with time. In contrast, the normal IgG behaved indifferently to the presence of tumour; its clearance from the tumour was similar to that of all the other tissues studied and its concentration was always lower than in blood (Fig. 4). Since the ratio of $^{125}$I-labelled LICR-LON/HT13 to $^{131}$I-labelled normal mouse IgG was the same in normal organs of the experimental animals and did not change appreciably with time, the distributions of the two antibodies in normal tissues appear similar. In our system, the specific antibody tumour/blood ratio was between 0·4 at 4 h and 10 at 96 h after injection, demonstrating a 2-fold (4 h) to a 14-fold (96 h) increase over the same ratio with normal IgG (Fig. 3a). Similarly, the specific antibody tumour/blood ratio showed a 3-fold (4 h) to a 20-fold (96 h) increase over the liver/blood ratio (Fig. 3c).

FIG. 6.—Chromatography profile (Sephacryl S-300) of $^{125}$I-labelled LICR-LON/HT13 in plasma of animals carrying HX39 human germ-cell tumour xenografts at 4, 24 and 38 h.

The localization described above could be inhibited by simultaneous i.v. injection of non-radiolabelled LICR-LON/HT13 (100 $\mu$g/animal), indicating that the cold antibody was capable of blocking the tumour antigenic sites, thus inhibiting uptake of further (radiolabelled) antibody by the tumour. In parallel experiments, such inhibition was not found when cold FIB-75 IgG was used.

If the localization observed is due to antibody–antigen reaction, it might be expected to occur rapidly after injection, and therefore selective homing of the antibody to the tumour should be detected by 4 h, when the first dissection of the animals took place. Indeed, it was found with the tumour/blood and tumour/non-tumour ratios with the monoclonal IgG were higher than the same ratios with the normal IgG 4 h after injection (Fig. 3b). Similarly, the specific-antibody tumour/blood ratio is superior to liver/blood ratio (Fig. 3c) as early as 4 h after injection. The same pattern is seen when the monoclonal antibody/normal IgG ratio in the tumour is compared to that in the blood (Fig. 3*a*).

Plasma samples of injected animals were passed through Sephacryl S-300 columns. The IgG eluted consistently at the expected fractions, with no peaks of radioactivity before or after the IgG peak, indicating that large molecules had not been formed and no degradation of antibody had taken place (Fig. 6). It was thought that shedding of antigen to the circulation from large tumours (which showed no uptake) may inhibit localization, as it would react with the injected antibody. However, the plasma profiles of large tumours (0·8–1·1 g) was the same as that of small tumours (8–86 mg).

Tumour weight was found to be important, in that the smaller the tumour the

FIG. 7.—The localization index of LICR-LON/HT13 in 3 different experiments (●, ■ and ▲) with tumours (HX39) of varying weights, dissected and counted 48 h after injection.

TABLE II.—*Localization of teratoma and non-teratoma xenografts with anti-teratoma antibody LICR-LON/HT13**

| Tumour | Pathology | LI† 48 h | LI† 96 h |
|---|---|---|---|
| HX39 | MTU‡ with yolk-sac elements | 7·0 | 10·9 |
| HX112 | Yolk-sac carcinoma | 6·5 | 7·7 |
| HX111 | MTU | 3·8 | 5·6 |
| HX53 | Seminoma with yolk-sac elements | 4·0 | 3·6 |
| HX57 | Yolk-sac carcinoma | 2·8 | 2·9 |
| HX99 | Breast adenocarcinoma | 1·1 | 1·1 |
| HX65 | Bronchial adenocarcinoma | 1·2 | 1·1 |
| HN.BR | Squamous-cell carcinoma | 1·6 | 1·4 |

* Mean of 4–8 tumours at each time. Tumour weight 15–22 mg.

‡ MTU—Malignant teratoma undifferentiated.

better the localization. No significant selective uptake of the antibody by the tumour was found in tumours weighing above 200–250 mg (Fig. 7). In single animals carrying 2 tumours of different weights, this difference was also noted (indicating that this phenomenon was tumour-dependent rather than animal-dependent).

The specificity of the localization of LICR-LON/HT13 for the germ-cell teratoma HX39/7 was tested in other tumours by using 4 additional germ-cell and 3 non-germ-cell xenografts. *In vivo* localization occurred in all the teratoma lines but not in the non-teratoma tumours (Table II). The degree of localization varied amongst the teratomas, but in none was it as high as for the HX39/7 tumour.

### DISCUSSION

This work demonstrates that a monoclonal antibody raised against a surface component of a human tumour xenograft is capable of selective localization in such a tumour after its *in vivo* administration. This was demonstrated as follows: (a) the uptake of the antibody by the tumour was higher than in blood and normal tissues, (b) localization did not occur with normal mouse IgG or an indifferent monoclonal antibody, (c) blocking of the antigen with excess antibody inhibited selective uptake and (d) preferential localization in the tumour was demonstrated not only with regard to normal mouse tissues and with normal IgG but also with regard to other tumours. The above findings support the hypothesis that the *in vivo* localization is due to the interaction of the injected antibody with the cell-surface antigen. Further evidence for this arises from preliminary work, where we have shown by autoradiography that, after i.v. administration, the monoclonal antibody is located in viable parts of the tumour and often seen to be closely applied to the periphery of individual HX39 teratoma cells (Moshakis *et al.*, 1981*b*).

The absence of immune complexes (large molecules eluting before the IgG peak) in the plasma of the animals after antibody injection suggests that the target cell-surface antigen does not reach the circulation. This, however, should not be taken as aiding localization, since similar work with antibodies to soluble tumour markers in patients and animals has shown that the presence of circulating antigen does not, surprisingly, have any relevance to the degree of localization (Goldenberg *et al.*, 1980; Moshakis *et al.*, 1981*a*).

Comparison of the concentration of the two radioisotopes in tumour, tissues and blood have made it possible to attribute the selective uptake of the antibody by the tumour to the binding properties of the antibody itself. Non-immunological factors that would affect the entry and handling of IgG by the tumour, such as vascularity, necrosis, lymphatic drainage and extracellular space, would apply equally to both specific and normal IgG. It appears that the technique of double-isotope labelling (Pressman *et al.*, 1957) still remains of great assistance in this field, especially in the experimental animal which can be killed and tissue radioactivity measured. This becomes more important when new reagents, such as monoclonal antibodies, are exploited for this purpose. Demonstration of localization in the animal model of human tumour xenografts lessens, but does not abolish, the need for such studies when this approach is used in patients, where tissue dissection is not often possible.

Correlation between *in vivo* localization and *in vitro* binding to cells was not possible when other teratomas were used, because none of the other 4 xenografts have been established in tissue culture. Nevertheless, the behaviour of the antibody and the normal IgG, together with the localization kinetics, were similar to the pattern seen when LICR-LON/HT13 used to localize the HX39 tumour. The specific uptake of the monoclonal antibody was highest in the HX39 tumour (against which the antibody was raised). This suggests that, although the antigen is present in all the germ-cell tumours examined, its "expression" was less in the other tumours than that which acted as an immunogen. Since the tumours examined contain varying amounts of yolk-sac carcinoma, seminoma and undifferentiated malignant teratoma, further studies are required, to correlate germ-cell tumour histology and site of antibody uptake at a cellular level. Such a study might reveal the reason for the varying degrees of localization of antibody among germ-cell tumours.

This study of tumour localization and the parameters which affect it, using a monoclonal antibody against a surface antigen on a human teratoma cell line, shows that such antibodies do localize *in vivo* and that they are, therefore, good candidates for clinical use, such as immunoscanning and tumour–drug targeting. In this respect, the property of the antibody to localize in small tumours could prove very useful in detecting micrometastases in patients, especially when the newer tomographic scanning techniques are fully developed. In the past, computerized background subtraction of the blood pool has been used in every case when conventionally raised antisera have been used to detect tumours in patients. However, the tumour/blood ratios obtained with monoclonal antibodies may make this technique unnecessary; Ballou *et al.* (1979) using a monoclonal antibody, have shown that murine tumours can be detected without background subtraction, if the scan is performed more than 48 h after antibody injection. It must be emphasized, however, that although the tumour/blood ratio increases with time the absolute amount of activity in the tumour decreases. The optimal time, therefore, for scanning is a balance between these two factors. It is interesting that our study, and those of others using monoclonal antibodies for localization (Ballou *et al.*, 1979; Houston *et al.*, 1980), demonstrate better tumour/blood and tumour/non-tumour ratios than those obtained with conventional antibodies to soluble tumour markers. However, it should not be forgotten that the antibodies used are of murine origin and injected into mice. Their value can only be assessed fully when these reagents are injected into patients.

### REFERENCES

BAGSHAWE, K. D., SEARLE, F., LEWIS, J., BROWN, P. & KEEP, P. (1980) Preliminary therapeutic and localisation studies with human chorionic gonadotrophin. *Cancer Res.*, **40**, 3016.

BAILEY, M. J., GAZET, J.-C. & PECKHAM, M. J. (1980) Human breast-carcinoma xenografts in immune-suppressed mice. *Br. J. Cancer*, **42**, 524.

BALLOU, B., LEVINE, G., HAKALA, R. J. & SOLTER, D. (1979) Tumour localisation detected with radioactivity labelled monoclonal antibody and external scintigraphy. *Science*, **206**, 844.

BEGENT, R. M. J., STANWAY, G., JONES, B. E. & 4 others (1980) Radioimmunolocalisation of tumours by external scintigraphy after administration of $^{131}I$ antibody to human chorionic gonadotrophin: Preliminary communication. *J.R. Soc. Med.*, **73**, 624.

DYKES, P. W., HINE, K. R., BRADWELL, A. R. & 4 others (1980) Localisation of tumour deposits by external scanning after injection of radiolabelled anti-carcinoembryonic antigen. *Br. Med. J.*, **281**, 220.

EDWARDS, P. A. W., FOSTER, C. S. & MCILHINNEY, R. A. J. (1980) Monoclonal antibodies to teratomas and breast. *Transplant. Proc.*, **12**, 398.

GALFRE, G., HOWE, S. C., MILSTEIN, C., BUTCHER, G. W. & HOWARD, J. C. (1977) Antibodies to major histocompatibility antigens produced by hybrid cell lines. *Nature*, **266**, 550.

GOLDENBERG, D. M., DELAND, F., KIM, E. & 6 others (1978) Use of radiolabelled antibodies to carcinoembryonic antigen for the detection and localisation of diverse cancers by external photoscanning. *N. Engl. J. Med.*, **298**, 1384.

GOLDENBERG, D. M., KIM, E., DELAND, F. & 6 others (1980) Clinical studies on the radioimmunodetection of tumours containing alpha-fetoprotein. *Cancer*, **45**, 2500.

GREENWOOD, F. C., HUNTER, W. M. & GLOVER, J. S. (1963) The preparation of $^{131}$I-labelled human growth hormone of high specific radioactivity. *Biochem. J.*, **89**, 114.

HOUSTON, L. L., NOWINSKI, R. C. & BERNSTEIN, I. D. (1980) Specific *in vivo* localisation of monoclonal antibodies directed against the Thy 1.1 antigen. *J. Immunol.*, **125**, 837.

KÖHLER, G. & MILSTEIN, C. (1975) Continuous cultures of fused cells secreting antibody of predefined specificity. *Nature*, **256**, 495.

MACH, J.-P., CARREL, S., MERENDA, C., SORDAT, B. & CEROTTINI, J. C. (1974) *In vivo* localisation of radiolabelled antibodies to carcinoembryonic antigen in human colon carcinoma grafted into nude mice. *Nature*, **248**, 704.

MACH, J.-P., CARREL, S., FORNI, M., RITSCHARD, J., DONATH, A. & ALBERTO, P. (1980*a*) Tumour localisation of radiolabelled antibodies against carcinoembryonic antigen in patients with carcinoma. *N. Engl. J. Med.*, **303**, 5.

MACH, J.-P., FORNI, M., RITSCHARD, J. & 5 others (1980*b*) Use and limitations of radiolabelled anti-CEA antibodies and their fragments for photoscanning detection of human colorectal carcinomas. *Oncodevelop. Biol. Med.*, **1**, 49.

MCILHINNEY, R. A. J. (1981) Cell surface molecules of human teratoma cell lines. In *Early Detection of Testicular Cancer*. (Ed Skakkebae *et al.*) Copenhagen: Scriptor. p. 93.

MOSHAKIS, V., BAILEY, M. J., ORMEROD, M. G., WESTWOOD, J. H. & NEVILLE, A. M. (1981*a*) Localization of human breast-carcinoma xenografts using antibodies to carcinoembryonic antigen (CEA). *Br. J. Cancer*, **43**, 575.

MOSHAKIS, V., MCILHINNEY, R. A. J., RAGHAVAN, D. & NEVILLE, A. M. (1981*b*) Monoclonal antibodies to detect human tumours: An experimental approach. *J. Clin. Pathol.*, **34**, 314.

PRESSMAN, D., DAY, E. D. & BLAU, M. (1957) The use of paired labelling in the determination of tumour-localising antibodies. *Cancer Res.*, **17**, 845.

PRIMUS, F. J., WANG, R. H., GOLDENBERG, D. M. & HANSEN, H. J. (1973) Localisation of human GW-39 tumours in hamsters by radiolabelled heterospecific antibody to carcinoembryonic antigen. *Cancer Res.*, **33**, 2977.

RAGHAVAN, D., GIBBS, J., HEYDERMAN, E., NEVILLE, A. M. & PECKHAM, M. J. (1980) Functional and morphological aspects of human teratoma xenografts. In *Thymus Aplastic Nude Mice and Rats in Clinical Oncology*. Ed. Bastert *et al.* Stuttgart: Fisher-Verlag.

SHORTHOUSE, A. J., SMYTH, J. F., STEEL, G. G., ELLISON, M., MILLS, J. & PECKHAM, M. J. (1980) The human tumour xenograft—a valid model in experimental chemotherapy? *Br. J. Surgery*, **67**, 715.

STEEL, G. G., COURTENAY, V. D. & ROSTOM, A. Y. (1978) Improved immune-suppression techniques for the xenografting of human tumours. *Br. J. Cancer*, **37**, 224.

WILLIAMS, A. F., GALFRE, G. & MILSTEIN, C. (1977) Analysis of cell surfaces by xenogenic myeloma-hybrid antibodies: Differentiation antigens of rat lymphocytes. *Cell*, **12**, 663.